# Health facility-related barriers associated with cervical cancer screenings in the health districts of Dakar and Kédougou (2023, Senegal)

**DOI:** 10.4102/jphia.v17i1.1879

**Published:** 2026-09-09

**Authors:** Fatoumata B. Diongue, Ibrahima Ndiaye, Ndeye M. Sougou, Oumar Bassoum, Caryn E. Peterson, Omar Gassama, Sarah Abboud, Fatou B. Seck, Malick Anne, Ndeye M. Dieng, Hilaire Sarr, Assane Sall, Adja M. Diaw, Aliath Salami, Aida Fall, Aida Barro, J. Andrew Dykens, Adama Faye

**Affiliations:** 1 Faculty of Medicine Pharmacy and Odontology, Cheikh Anta Diop University, Dakar, Senegal; 2 Division of Epidemiology and Biostatistics, School of Public Health, University of Illinois Chicago, Chicago, Illinois, United States of America; 3 Center for Global Health, College of Medicine, University of Illinois Chicago, Chicago, Illinois, United States of America; 4 Cancer Center, University of Illinois Chicago, Chicago, Illinois, United States of America; 5 Department of Obstetrics and Gynecology, Aristide Le Dantec Teaching Hospitals, Cheikh Anta Diop University, Dakar, Senegal; 6 Center for Training in Women’s Health, Cheikh Anta Diop University, Dakar, Senegal; 7 Department of Human Development Nursing Science, College of Nursing, University of Illinois Chicago, Chicago, Illinois, United States of America; 8 Office of Cancer Prevention and Control, Division of Non-Communicable Diseases, Ministry of Health and Public Hygiene, Dakar, Senegal; 9 Department of Family and Community Medicine, University of Illinois Chicago, Chicago, Illinois, United States of America

**Keywords:** barriers, cervical tumours, early cancer screening, health facilities, Senegal

## Abstract

**Background:**

Because of low utilisation of cervical cancer screening (CCS) services, Senegal ranks 17th in the world for cervical cancer incidence.

**Aim:**

The aim was to investigate health facility factors associated with CCS.

**Setting:**

The study was conducted in six Senegalese health districts (Keur Massar, Yeumbeul, Diamniadio, Kédougou, Saraya and Salémata).

**Methods:**

This was a descriptive and analytical cross-sectional study of women aged 25–69 years. A two-stage cluster survey was carried out, with a selection of census districts (CDs) followed by households. The data were collected with collection tools during individual interviews. Data were analysed using R 4.2.3 software. Univariate, bivariate analysis and logistic regression were performed.

**Results:**

The study involved 360 women. The average age was 40.2 (± 11.5) years. A proportion of 46.5% (334 individuals) regularly went to the health facility for treatment. 18.8% of women had been screened for cervical cancer. Factors associated with screening were hearing about a CCS or the human papillomavirus vaccine, regular attendance at a traditional healer for care, regular attendance at a healthcare facility to receive care and professionalism of healthcare facility workers. Other significantly related factors were age, marital status and level of education.

**Conclusion:**

Improving the uptake of CCS services requires a multifactorial approach, including taking into account obstacles linked to health facilities. It will involve improving attendance at health facilities through information, education and communication strategies aimed at women and health workers to contribute to maternal health.

**Contribution:**

This study will help remove the barriers faced by healthcare facilities that limit screening, including a lack of information, reliance on traditional medicine, irregular visits to healthcare facilities, and a lack of professionalism among healthcare workers. As a result, an improvement will be observed in the utilisation of cervical cancer screening services. Policy makers will be able to use the results to implement appropriate strategies that will increase screening rates and ensure adequate care for women who test positive. This study makes a positive contribution to science and opens up avenues for further research, such as on the implementation of cervical cancer screening services.

## Introduction

Cervical cancer is the most common malignancy in women, caused by unresolved long-term infection with distinct genotypes of human papillomavirus (HPV).^1^ It is the fourth most common cancer neoplasia in women worldwide.^2^ The two oncogenic genotypes, HPV 16 and 18, are responsible for over 70% of cervical cancers worldwide.^1^ Cervical cancer remains a major public health problem worldwide.^3,4^ It is the most common gynaecological cancer in developing countries.^1^ It is a preventable cancer but is also widespread in many low- and middle-income countries (LMICs), including sub-Saharan Africa (SSA),^5^ South Africa^6^ and Ethiopia,^7^ among others.

Cervical cancer continues to represent a significant burden of morbidity and mortality in LMICs.^8^ It is the leading cause of cancer death among women in 37 countries, mainly in SSA,^9^ the second leading cause of cancer death and one of the 20 leading causes of death among women in Ethiopia.^7,10,11^ It ranks among the top five cancers in terms of incidence and mortality in LMICs.^12^ Every year, over 650 000 women worldwide are diagnosed with cervical cancer, and more than half of them succumb to the disease.^6,13,14^ Lack of awareness and insufficient access to early detection and pre-cancer treatment contribute to high mortality.^8^ Less than one in six eligible girls is vaccinated against HPV worldwide.^9^ Cancer, as a whole, has a negative impact on the economic growth of countries^15^ and is a major public health problem, responsible for nearly one in six (16.8%) and one in four (22.8%) deaths from non-communicable diseases (NCDs) worldwide.^16^ The disease causes enormous pain and suffering for the individual and has significant negative effects on the well-being of their family and community.^17^

In 2022, around 1800 women were diagnosed with cervical cancer and nearly 1000 women died from the disease worldwide.^9^ A study on the integration of opportunistic screening into primary healthcare conducted in Dakar between 2018 and 2021 showed a visual inspection with acetic acid (VIA) positivity of 3.5% (Selmouni 2022).^18^

Cervical cancer is one of the easiest forms of cancer to prevent and treat if detected early. Primary cytology screening and molecular HPV testing are important for cervical cancer prevention.^1^ Only 36% of women worldwide have been screened for cervical cancer, with major disparities from one country to another (4% in Ethiopia), with almost 100% of women in Sweden and the Netherlands.^9^ In developed countries, over 60% of women have been screened for cervical cancer. However, in SSA, only 12% of women have been screened for precancerous cervical lesions.^19^

More than 80% of cervical cancer cases in SSA are detected at late stages, mainly because of the absence or inaccessibility of preventive services.^20^ Largely because of low uptake of cervical cancer screening (CCS) services,^21^ Senegal ranks 17th worldwide for cervical cancer incidence.^22^ For women aged 18–69 years in Senegal, the CCS rate is very low (6.9%).^21,23^ For women aged from 40 years to 49 years in rural areas of the country, the screening rate is less than 2%.^23^ As a result, women often present with advanced cancers. Lessons from higher-resource countries show that incidence and mortality rates are falling, and that premature deaths could be avoided through early detection, follow-up and appropriate treatment.^22^

To improve screening rates, it is helpful to understand the factors that contribute to lower screening rates in various contexts.^3,4^ Age, place of residence, education, occupation, level of knowledge about cervical cancer and screening and attitude towards CCS are important determinants of the uptake of CCS. Other factors include personal barriers such as fear, lack of knowledge, cultural beliefs and women’s role as caregivers. Systemic barriers include communication problems, shortage of primary care providers and negative health experiences. Motivating factors that prompt women to undergo screening may include a family history of cancer, encouragement from providers and reminders from clinics.^4^ Taking these factors into account when developing policies and programmes could improve uptake of CCS.^4,10^

This research focuses on the barriers to screening that result in under-utilisation of screening services. While many studies focus on global factors in CCS, few studies focus specifically on healthcare facilities. This research focuses on the barriers to screening that result in under-utilisation of CCS services in order to further inform the shaping of health policies, the management of health facility-level barriers to screening and, ultimately, to improve screening rates. Hence, this study aims to investigate the barriers to CCS in healthcare facilities in various contexts in Senegal, the Dakar (urban and semi-urban) and Kédougou (rural) regions in 2023.

## Research methods and design

### Setting section and a map

The survey and intervention zones were three health districts of the Dakar health regional direction, namely Keur Massar, Yeumbeul and Diamniadio and three districts of the Kédougou health regional direction, namely Saraya, Salémata and Kédougou. The selection of the six districts was through convenience sampling and is representative of Senegal’s urban and rural contexts while supporting ready access to CCS services, local treatment for pre-cancer and available referral for advanced diagnostics and treatment. In each district, the health centre and two health posts Poste de Santé [PS] were considered. In Dakar, the health posts were Mme Fatou Ba, Boune, Yeumbeul Diamalaye, Maternity of Yeumbeul Nord, Niangal and Sebikotane, while in Kédougou, the health posts were Bembou, Diakha Macky, Dimboli, Fongolimby, Darou Ningou and Darou Salam^24^ (Figure 1).

**FIGURE 1:**
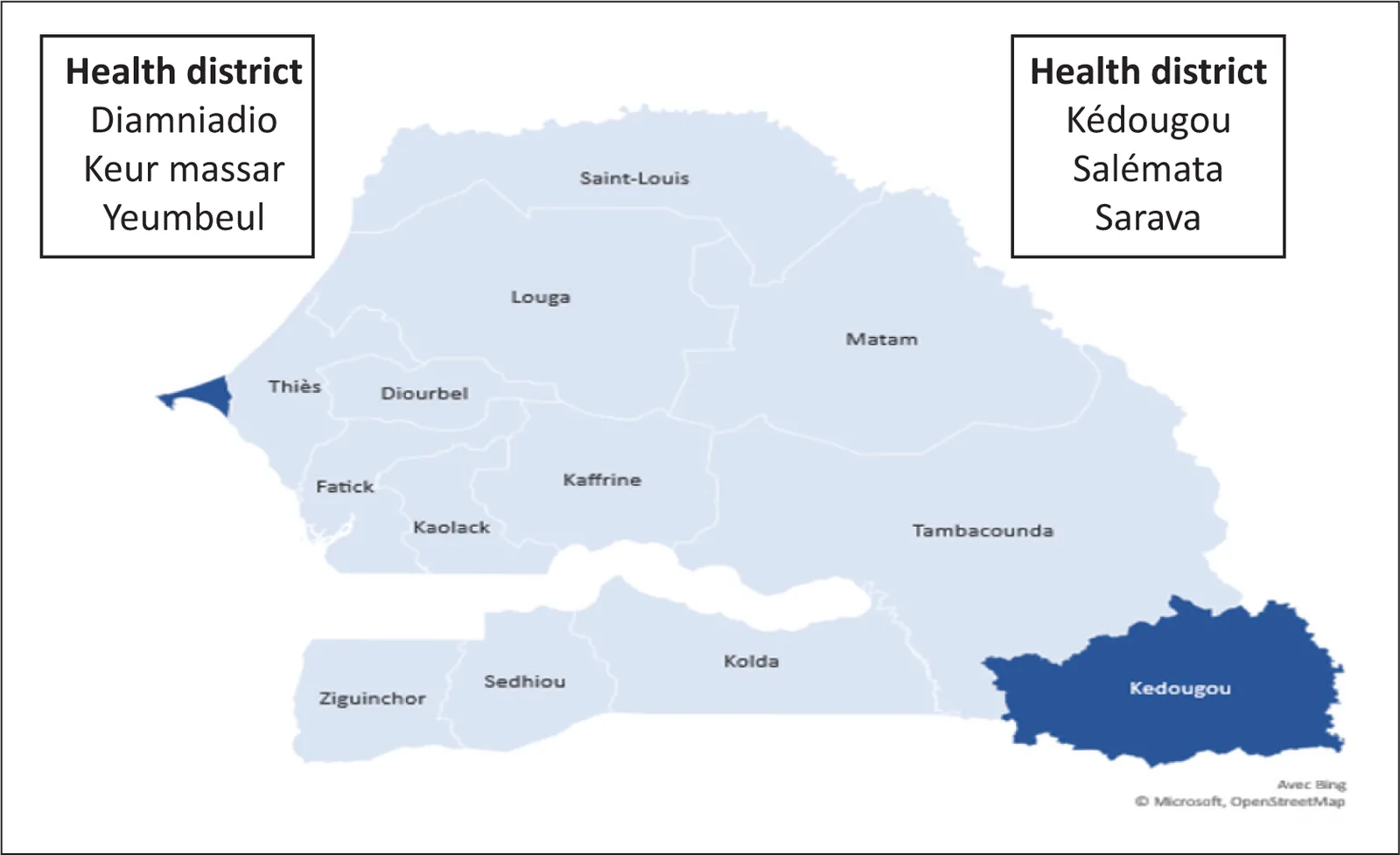
Six districts selected through convenience sampling and representative of Senegal’s urban and rural contexts.

### Methodology

This is a descriptive and analytical cross-sectional study. In each health district (cluster), the reference health centre and two randomly selected health posts were included.

The study population consisted of women aged 25–69 years. Included were women eligible for CCS and men living in a household with at least one woman eligible for CCS in a healthcare facility.

A two-stage cluster survey was used to select the clients to be interviewed. At the first level, primary units were census districts (CDs). A CD is a geographical area with precise boundaries and a certain number of concessions made up of households. The CDs were selected by random selection. Three CDs were selected within the health centre area, and two CDs at the level of each post were considered. In the second stage, in each selected CD, a mapping exercise was carried out, with the help of neighbourhood leaders, to identify households with at least one woman eligible for CCS between 25 years and 69 years old. In the CDs, the first household was randomly selected. Subsequent households were selected by systematic random sampling. Households were drawn in each health centre’s CDs for a total of 30 women. In the health posts, 10 households were selected in one CD and five in another CD for a total of 15 women per health post. Systematic sampling ensured equal proportions within the enumerated population.

Data were collected from 10 October 2023 to 19 March 2024, using questionnaires administered by trained research staff in the preferred language (French, Malinké or Pulaar) of participants. A total of three questionnaires were drawn up for the female target groups, with the following information collected: (1) Consent: Participant eligibility, participant signature and information, consent obtained by research personnel and witnessing the consent process; (2) demographics and knowledge and (3) healthcare facility data: Barriers to healthcare facilities (utilisation, quality of care and trust). Interviewers were selected on the basis of their ability to communicate, their level of education and their knowledge of national languages. Field agents underwent a 2-day training course, covering all aspects of the survey and the use of tablets and the REDCap application for data collection. The training was followed by a 1-day pre-test in a location other than the survey site. Data were collected during face-to-face interviews for all participants. Questions were asked by the interviewers in the participant’s preferred language (French, Wolof, Malinké or Pulaar).

Data quality control was carried out at several levels. Each of the questionnaires was tested between the target groups before collection began, in order to standardise them. Debriefing meetings were held at the end of each day to discuss any difficulties, and the team leader checked all questionnaires to ensure quality completion before sending them to the server. Supervisors regularly checked the server to see how far the collection had progressed. The application used to collect data was developed using REDCap software. This application was used to design the data entry mask and offered the possibility of collecting and transferring the data to a server created at the University of Illinois at Chicago. The advantage of this software is that it not only reduces design time but also data entry errors. This is because of its ability to integrate controls at the question level, notably not only limits for certain quantitative variables but also filters. To ensure good data quality, certain functionalities are implemented in the data collection application, notably range limits and filters. Once the questionnaire has been administered, the interviewer carries out a check to ensure the quality of the responses. Finally, a check on data structure and consistency is carried out during the audit and correction phase at the end of data collection.

Data analysis, using R 4.2.3 software, involved variable description, bivariate and multivariate analysis with binomial logistic regression. The automated top-down stepwise method was used. We built the full model using variables with *p*-values less than 0.25. The model with the lowest Akaike information criterion (AIC) was selected. Validation criteria for the final model included multi-collinearity and model calibration using the Hosmer–Lemeshow test. The parameter measured was the adjusted odds ratio (OR) and its 95% confidence interval.

### Ethical considerations

Ethical clearance to conduct this study was obtained from the University of Illinois Chicago Cancer Center Protocol Review Committee (No. 2022-0055), the University of Illinois Chicago Institutional Review Board (No. 2021-1282) and the National Ethics Committee for Health Research at the Senegal Ministry of Health and Public Hygiene (No. SEN22/133). All project staff were asked to sign a confidentiality agreement throughout data collection. Survey data were automatically stored in the secure REDCap database. Only the research team had access to private information. Prior to the start of the study, informed consent was obtained from all participants by means of a written consent procedure in a language that the participant could understand. The interviews took place in a secluded location, away from prying eyes. This project is part of a patient navigation programme^24^ and is guided by a research partnership comprising the Senegal Ministry of Health and Social Action, Cheikh Anta Diop University (UCAD) in Dakar, and the University of Illinois Chicago (UIC).

## Results

### Characteristics description

#### Sociodemographic characteristics

The study involved 360 women with mean age for women of 36.9 (± 10.2) years. Of these, 92.5% were married, 75.5% had primary, Koranic education or no education, and 24.5% had secondary, higher education or professional training (Table 1).

**TABLE 1:** Distribution of individuals by sociodemographic characteristics, knowledge and factors related to healthcare facilities.

Features	Terms and conditions	Frequency	
		*n*	%
Marital status	Married	333	92.5
	Not married	27	7.5
Highest level of education achieved	No-primary level-Koraniq teaching	271	75.5
	Secondary, superior level or professional	88	24.5
Hearing about cervical cancer	No	85	23.6
	Yes	275	76.4
Hearing about a cervical cancer screening test	No	126	35.0
	Yes	234	65.0
Hearing about the human papillomavirus (HPV)	No	252	70.0
	Yes	108	30.0
Hearing about the HPV vaccine	No	211	59.3
	Yes	145	40.7
I regularly go to a traditional healer for treatment	Agreement	133	37.3
	Other	224	62.7
I regularly go to the health centre for treatment	Agreement	290	80.8
	Other	69	19.2
I go to the health structure to avoid any medical problems, even though I’m fine at the moment	Agreement	203	57.2
	Other	152	42.8
I would be in favour of taking a vaccine to protect myself from a medical problem	Agreement	300	83.6
	Other	59	16.4
In the past year, have you had your blood pressure checked by a doctor, nurse or other health professional?	ne sait pas-pas sur	6	1.7
	No	130	36.1
	Yes	224	62.2
Have you been tested for HIV in the last 5 years?	Don’t know – not sure	24	6.7
	No	221	61.6
	Yes	114	31.8
It’s easy to see my healthcare provider to receive the care I need	Agreement	288	80.7
	Other	69	19.3
My healthcare provider is too far away for me to see him regularly	Agreement	166	46.4
	Other	192	53.6
The healthcare facility where I receive my care is comfortable and welcoming	Agreement	321	89.2
	Other	39	10.8
I don’t have to wait long at the healthcare facility to see the healthcare provider	Agreement	234	65.4
	Other	124	34.6
I feel comfortable with my healthcare provider	Agreement	319	89.6
	Other	37	10.4
My healthcare provider has the time to take care of all my needs	Agreement	316	90.0
	Other	35	10.0
I always receive all my healthcare in a language I understand perfectly	Agreement	311	88.9
	Other	39	11.1
The equipment, materials and supplies of the healthcare facility are adequate and clean	Agreement	271	78.1
	Other	76	21.9
I am able to speak with my supplier in private	Agreement	319	92.7
	Other	25	7.3
When my supplier performs a physical examination, he is completely private	Agreement	306	89.2
	Other	37	10.8
I receive regular and appropriate information and explanations about my state of health and my care	Agreement	243	71.1
	Other	99	28.9
My healthcare facility helps me book appointments and follow-up care	Agreement	267	79.0
	Other	71	21.0
Sometimes I’ve been treated badly when receiving healthcare	Agreement	58	16.2
	Other	301	83.8
Patients have sometimes been deceived or misled in the healthcare establishment	Agreement	48	13.4
	Other	310	86.6
Healthcare establishments have sometimes carried out harmful experiments on patients without their knowledge	Agreement	51	14.2
	Other	309	85.8
Healthcare facilities put patients’ health first	Agreement	290	81.0
	Other	68	19.0
Patients should always follow the advice given to them in the healthcare facility	Agreement	323	90.5
	Other	34	9.5
The healthcare facility’s agents are professionals	Agreement	312	87.6
	Other	44	12.4
Errors are common in the healthcare establishment	Agreement	100	28.1
	Other	256	71.9
Screened for cervical cancer	No	290	81.2
	Yes	67	18.8

### Awareness knowledge

Of those surveyed, 76.4% had heard of cervical cancer, 65.0% of a CCS test, 30.0% of the HPV and 40.7% of the HPV vaccine (Table 1).

### Characteristics related to healthcare facilities

#### Use of health facilities and preventive care

Among the targets, 37.3% regularly went to a traditional healer for treatment and 80.8% to a health facility for treatment. Some (57.2%) visited the facilities even in the absence of signs to avoid medical problems. Others (83.6%) would be in favour of taking a vaccine to protect themselves against a medical problem. Only 62.2% of respondents have checked their blood pressure with a doctor, nurse or other health professional in the last year. Over the last 5 years, 31.8% have been tested for human immunodeficiency virus (HIV) (Table 1).

#### Quality of care in healthcare facilities

Several languages are used in health facilities by healthcare providers with the targets, the majority being Wolof (50.0%), followed by Pulaar (25.0%) and Malinké (19.7%). Respondents (80.7%) mentioned the ease of seeing the healthcare provider to receive necessary care. A proportion of 46.4% mentioned the remoteness of the healthcare provider for a regular visit, 89.2% the comfort and welcome of the health facility and 65.4% the shorter waiting time. Respondents also mentioned the feeling of being at ease with the healthcare provider (89.6%), the consideration given to all individual needs (90.0%), the care received in a language fully understood (88.9%), the adequacy and cleanliness of the healthcare facility’s equipment and materials and supplies 78.1%. Some (92.7%) were able to talk to the provider in private, and physical examinations (89.2%) were done in private. Most (71.1%) regularly received adequate information and explanations about their health and care, and 79.0% booked appointments and followed up on care through the health facility (Table 1).

### Trust in healthcare structures

Few people (16.2%) talked about abuse in healthcare. Sometimes they are deceived or misled in healthcare facilities (13.4%). Some (14.2%) believe that facilities have sometimes carried out harmful experiments on patients without their knowledge; others, on the other hand, believe that healthcare facilities give priority to the patient’s health (81.0%) and patients must always follow the advice given to them in the healthcare facility (90.5%). A large number spoke of healthcare workers being considered professionals (87.6%), but those errors were frequent at the facility level (28.1%) (Table 1).

### Cervical cancer screening

Among women, only 18.8% have ever been screened for cervical cancer.

### Bivariate analysis

The women screened were older (40.8 ± 12.0 vs. 35.9 ± 9.5). Women in secondary school, higher education or vocational training were more likely to be screened. The proportion of women screened was higher in Dakar than in Kédougou. Women who had heard of cervical cancer, the CCS test, the HPV and the HPV vaccine were more likely to be screened (Table 2).

**TABLE 2:** Status and knowledge about cervical cancer associated with cervical cancer screening.

Features	Terms and conditions	Screening				*p*-value
		No		Yes		
		*n*	%	*n*	%	
Preferred method of contact	-	-	-	-	-	0.37
	Phone	212	75.7	54	81.8	-
	Home visit	68	24.3	12	18.2	-
Marital status	-	-	-	-	-	0.191
	Married	266	91.7	65	97.0	-
	Unmarried	24	8.3	2	3.0	-
Highest level of education achieved	-	-	-	-	-	0.005*
	Quranic school	227	78.5	41	61.2	-
	Secondary-superior-vocational	62	21.5	26	38.8	-
Region	-	-	-	-	-	< 0.001*
	Dakar	127	43.8	52	77.6	-
	Kédougou	163	56.2	15	22.4	-
Heard of cervical cancer	-		-	-	-	< 0.001*
	No	84	29.0	1	1.5	-
	Yes	206	71.0	66	98.5	-
Heard of a cervical cancer screening test	-	-	-	-	-	< 0.001*
	No	123	42.4	2	3.0	-
	Yes	167	57.6	65	97.0	-
Heard of the human papillomavirus (HPV)	-	-	-	-	-	< 0.001*
	No	215	74.1	34	50.7	-
	Yes	75	25.9	33	49.3	-
Heard of the HPV vaccine	-	-	-	-	-	< 0.001*
	No	187	65.4	21	31.3	-
	Yes	99	34.6	46	68.7	-

* , Significant *p*-value.

The screening rate was lower among women who agreed to go regularly to a traditional healer for care, and those who said their healthcare provider was too far away for them to see him regularly. It was higher among those who agreed with going regularly to the health facility for care. Women who always received care in a language they understood perfectly carried out more screening tests. However, this relationship was not significant (*p* = 0.09). Among the women who were more compliant with CCS were those who regularly received adequate information and explanations about their health and care, those who agreed that the advice given to patients in healthcare facilities should be followed and those who believed that healthcare workers in the healthcare facility were professionals. Women who agreed with these statements were more or less committed to screening. These factors were regular attendance at a traditional healer to receive care (crude odds ratio [ORb] = 0.53 [0.29–0.96]); regular attendance at a health facility to receive care ORb = 4.54 (1.61–12.5); distance from the healthcare provider for regular visits (ORb = 0.47 [0.27–0.84]); the regular receipt adequate information and explanations about health status and care (ORb = 2.22 [1.11–4.54]); following the advice given to patients in healthcare facilities (ORb = 8.33 [1.15–50.0]); the professionalism of healthcare workers in healthcare facilities (ORb = 11.11 [1.54–100.0]). Receipt of healthcare always in a perfectly understood language was associated but not significantly (crude odds ratio [ORb] = 3.03 [0.90–10.0]) (Table 3).

**TABLE 3:** Association between healthcare facility factors and cervical cancer screening (ORb [95% CI]).

Features	ORb	95% CI	*p*-value
Regularly go to a traditional healer for treatment: Agreement vs. other	0.53	0.29–0.96	0.038
Regularly go to the health facility for treatment: Agreement vs. other	4.54	1.61–12.5	0.004
Healthcare provider too far away to see him regularly. Agreement vs. other	0.47	0.27–0.84	0.010
Regularly receive adequate information and explanations about my health and healthcare: Agreement vs. other	2.22	1.11–4.54	0.024
Patients should always follow the advice given to them in the healthcare facility. Agreement vs. other	8.33	1.15–50. 0	0.036
The healthcare facility’s agents are professionals. Agreement vs. other	11.11	1.54–100.0	0.017
Go to the health facility to prevent a medical problem from occurring, even if I am currently well. Agreement vs. other	0.71	0.41–1.23	0.216
Always receive all my healthcare in a language I understand perfectly. Agreement vs. Other	3.03	0.90–10.0	0.074

ORb, crude odds ratio; CI, confidence interval.

### Multivariate analysis

Women who were older or married, with a high level of education from secondary school upwards, who had already heard of a CCS test or the HPV vaccine or who regularly went to the health structure for care, were more likely to be screened. On the other hand, the opposite situation was observed among those who regularly went to a traditional healer for care. Furthermore, the professionalism of health workers had a positive effect on women’s subsequent use of screening (Table 4).

**TABLE 4:** Health facility factors associated with cervical cancer screening by modelling.

Features	Crude OR	95% CI	Adj. OR	95% CI	Sig.
Age (years)	1.04	1.02–1.07	1.07	1.03–1.1	*
Marital status: Unmarried vs. married	0.34	0.08–1.48	0.14	0.03–0.72	*
Level of education: Second-highest-prof vs. no-primary-coranic	2.32	1.32–4.09	3.36	1.57–7.21	*
Heard of a cervical cancer screening test: Yes vs. no	23.94	5.75–99.61	12.17	2.6–56.92	*
Heard of the human papillomavirus (HPV) vaccine: Yes vs. no	4.14	2.34–7.32	2.05	1.03–4.1	*
Regularly go to a traditional healer for treatment: Other vs. Agreement	1.89	1.04–3.44	2.13	1.02–4.46	*
Regularly go to the health facility for treatment: Other vs. Agreement	0.22	0.08–0.62	0.23	0.07–0.76	*
Always receive all my healthcare in a language I fully understand: Other vs. Agreement	0.33	0.1–1.11	3.68	0.75–18.09	-
The healthcare facility’s agents are professionals: Other vs. Agreement	0.09	0.01–0.65	0.14	0.02–1.22	-
Regularly receive adequate information and explanations about my health and healthcare: Other vs. Agreement	0.45	0.22–0.9	0.62	0.27–1.44	-

Sig., significant; OR, odds ratio; CI, confidence interval; Adj., adjusted.

* , Statistically significant test.

## Discussion

This study has several strengths, such as the use of a two-stage cluster sampling method and the inclusion of variables related to the use of screening services. Furthermore, the relatively large sample size and the use of multivariate analysis, which helped to account for confounding factors, strengthen the validity of the results. However, a number of limitations are noted, such as the cross-sectional study design and the distribution of the sample across six districts. Additionally, some data are self-reported, which may introduce social desirability and recall biases.

The study found that 18.6% of women had undergone CCS. Factors associated with screening were age, marital status, level of education and healthcare facility-related factors, including hearing about a CCS test or the HPV vaccine, regular attendance at a healthcare facility or traditional healer for care and the professionalism of healthcare facility workers.

In our study, 18.6% of women were screened for cervical cancer. These results are similar to those of Jemal et al., who found that, worldwide, a significant proportion of women make little use of CCS services (only 19.6% screened).^25^ Yimer et al. found a lower rate of 12.87% in SSA^26^ and in Ethiopia 4%.^26^ Our results are below those of the American Cancer Society, where only 36% of women worldwide have been screened for cervical cancer, with significant disparities from one country to another. In Sweden and the Netherlands, nearly 100% of women eligible for screening have undergone it.^9^ Even in nursing cohorts, the CCS rate remains low at 27.7%. Despite being considered a disease of public health importance by 74.0% of nurses,^6^ Lawson et al. found 81% among those who received VIA as their first screening, 19% HPV deoxyribonucleic acid (DNA) testing and less than 1% receiving both at the same time.^8^ Yasin et al. also found low screening at 30.3% among HIV-positive women.^11^ This difference in screening rates may be explained by the importance attached to this disease or by ignorance or misunderstanding of the disease. Cervical cancer continues to represent a significant burden of morbidity and mortality in LMICs. Strategies must therefore be undertaken to increase screening rates,^8^ such as structured population-based programmes to achieve the goal of eliminating cervical cancer.^27^ Decentralisation of the screening programme and intensification of efforts at primary and secondary care levels are needed to increase screening performance.^28^

Our study found a mean age of 36.9 (± 10.2) years among women, and that age is a predictive factor (adjusted odds ratio [ORa] = 1.07 [1.03–1.1]). The older you get, the more likely you are to be screened for cervical cancer. These results concur with those of these authors. For Ezzelarab et al., the average age of women at the time of screening was 38.5 years (± 10.5).^27^ According to Yimer et al., age is a predictive factor.^26^ In a study by Legasu et al., age was a significant determinant of CCS uptake.^7^ Gebremeskel et al. also demonstrated belonging to the 18–30 years age group (ORa = 0.46 [0.22–0.98]) as a determinant of CCS uptake among HIV-positive women.^10^ Assefa et al. concurred, stating that the 35+ age group is a determinant of CCS uptake.^17^

The present study found that higher levels of education from secondary onwards increased the likelihood of screening (ORa = 3.36 [1.57–7.21]). These results confirm those of Legasu, where secondary education is a significant determinant of the use of CCS services,^7^ and Yimer et al.^26^ and Jemal et al. showed that having a degree (ORa = 0.48 [0.24–0.98] was statistically significantly associated with the use of CCS.^25^ Gebremeskel et al. also demonstrated a lack of formal education (ORa = 0.25 [0.14–0.45]) and primary education (ORa = 0.28 [0.16–0.49]), as determinants of CCS uptake among HIV-infected women.^10^ Heinert et al. demonstrated the delay in CCS, which was significantly higher in patients who did not have a high school diploma (*p* = 0.01).^29^

The present study found that 76.4% of those surveyed had heard of cervical cancer, 65.0% had heard of a CCS test, 30.0% had heard of the HPV and 40.7% had heard of the HPV vaccine. A survey of healthcare workers, in this case nurses, showed that the majority had insufficient knowledge about cervical cancer and screening. Among them, 16.0% thought that the cervical cancer vaccine should not be given to girls under 16 years.^6^

Our study found that hearing about a CCS test (ORa = 12.17 [2.6–56.92]) increased the likelihood of screening, as did hearing about the HPV vaccine (ORa = 2.05 [1.03–4.1]). Some authors have demonstrated a significant link between knowledge of the disease and use of screening services. This is the case of Yasin et al., who found that women who were knowledgeable about cervical cancer were 2.54 times more likely to benefit from CCS than those who were uninformed (ORa = 2.54 [1.42–4.56]).^11^ Yimer et al., in a meta-analysis of seven studies, showed that knowledge of cervical cancer increased screening uptake by a factor of almost five (odds ratio [OR] = 4.81 [3.06–7.54]), and knowledge of screening sites was also a predictive factor.^26^ Good knowledge of CCS is a significant determinant of the use of CCS services, according to Legasu et al.^7^ Gebremeskel et al. also demonstrated low knowledge of cervical cancer as a determinant of CCS uptake among HIV-infected women.^10^ Jemal et al. also demonstrated that knowledge of CCS was associated with CCS uptake.^25^ Lack of awareness of screening recommendations is a barrier to CCS.^29,30^ To improve uptake of screening, healthcare professionals should strive to raise awareness of cervical cancer, improve attitudes towards CCS and advance knowledge of the disease. The collaboration of healthcare providers with governmental and non-governmental stakeholders plays a crucial role in addressing issues of accessibility and uptake of screening services.^11^ Chitha et al. have highlighted nurses’ lack of knowledge about cervical cancer and negative attitudes towards the HPV vaccine and screening, issues that the healthcare system needs to address urgently. A number of patient and healthcare system barriers affect participation in CCS. All these challenges require a systemic approach to nurse training, capacity building and health system strengthening.^6^ In addition, community uptake of HPV vaccines can only be improved if the attitudes of nurses and other healthcare workers improve, as they will advocate for the uptake of these vaccines by community members. There is a need to improve nurses’ attitudes towards the HPV vaccine and to remove barriers to CCS faced by patients and health systems.^6^

In our study, regular attendance at a healthcare facility for treatment (ORa = 4.34 [1.31–14.28]) increased screening uptake by more than four-fold. It would therefore be advisable to encourage the use of healthcare facilities. In some countries, however, most healthcare facilities do not offer CCS.^31^ According to Heinert et al., the delay in CCS was significantly higher among patients who had not had a routine examination in the previous 5 years (*p* = 0.01).^29^ Obstacles to low uptake of screening are highlighted, including lack of health education materials, limitation of service to a specific area, interruption of service, provider incompetence, lack of trust and lack of attention from the trained provider. Contextualised discussions and health promotion through training focused on low levels of knowledge, low levels of education, and the availability of CCS services are essential.^25^

In our study, regular attendance of a traditional healer for care (ORa = 0.46 [0.22–0.98]) decreased the use of screening. Chitha et al. found reasons, such as belief in traditional medicine, given by patients for not returning for their CCS results.^6^ The attitude of healthcare professionals influences women’s behaviour. Indeed, our study showed that the professionalism of healthcare workers in healthcare facilities is a factor contributing to screening (ORb = 11.11 [1.53–100]). Screening was less observed among women who said their healthcare provider was too far away for them to see him regularly. The healthcare provider’s remoteness for regular visits (ORb = 0.47 [0.27–0.84]) is a determinant of the use of care. One of the reasons given by patients for not returning for their CCS results was lack of transport.^6^

Women who regularly received adequate information and explanations about their health status and care, as well as those who agreed to follow the advice given in healthcare facilities to patients, carried out more screening (ORb = 2.22 [1.11–4.54] and ORb = 8.33 [1.15–50.0], respectively). One strategy for improving CCS is to integrate it into family planning centres, which offers great potential for reaching large numbers of women of childbearing age.^5^ Some strategies are considered effective. These include removing barriers, promoting motivating factors and widening access to screening services to reduce the number of cases and deaths because of breast and cervical cancer.^4^

## Conclusion

The uptake of CCS by women was low at 18.6%. Factors associated with screening were age, marital status, level of education and factors related to health facilities, including hearing about a CCS test or the HPV vaccine, regular attendance at a health facility or traditional healer for care and the professionalism of health workers at health facilities. Participation in CCS is low in the districts studied and influenced by several factors. The same situation is observed in SSA. Taking these factors into account when developing policies and programmes could improve the uptake of CCS at district and national levels. It is necessary to strengthen the information and communication policy, educate girls and, above all, review the attitude of health workers to improve the reception and setting in order to increase the use of screening services. Awareness-raising and health promotion targeting the predictive factors identified are important to increase the use of screening services.
